# Supplementary material for: Structural heterogeneity-induced enhancement of transverse magneto-thermoelectric conversion revealed by thermoelectric imaging in functionally graded materials
Source: Sci Technol Adv Mater. 2026 Mar 12;27(1):2643965. doi: 10.1080/14686996.2026.2643965 (PMC13040573; doi:10.1080/14686996.2026.2643965)
Supplement: Supplemental Material [file TSTA_A_2643965_SM1655.pdf]

Supplementary Information for

Structural heterogeneity-induced enhancement of transverse magneto-thermoelectric conversion revealed by thermoelectric imaging in functionally graded materials

Sang J. Park[1,*], Ravi Gautam[1], Takashi Yagi[2], Rajkumar Modak[3], Hossein Sepehri-Amin[1] and Ken-ichi Uchida[1,3,*]

[1] National Institute for Materials Science, Tsukuba 305-0047, Japan

[2] National Institute of Advanced Industrial Science and Technology, Tsukuba 305-8563, Japan

[3] Department of Advanced Materials Science, Graduate School of Frontier Sciences, The University of Tokyo, Kashiwa 277-8561, Japan

*Correspondence to: PARK.SangJun@nims.go.jp (S.J.P.); UCHIDA.Kenichi@nims.go.jp (K.U.)

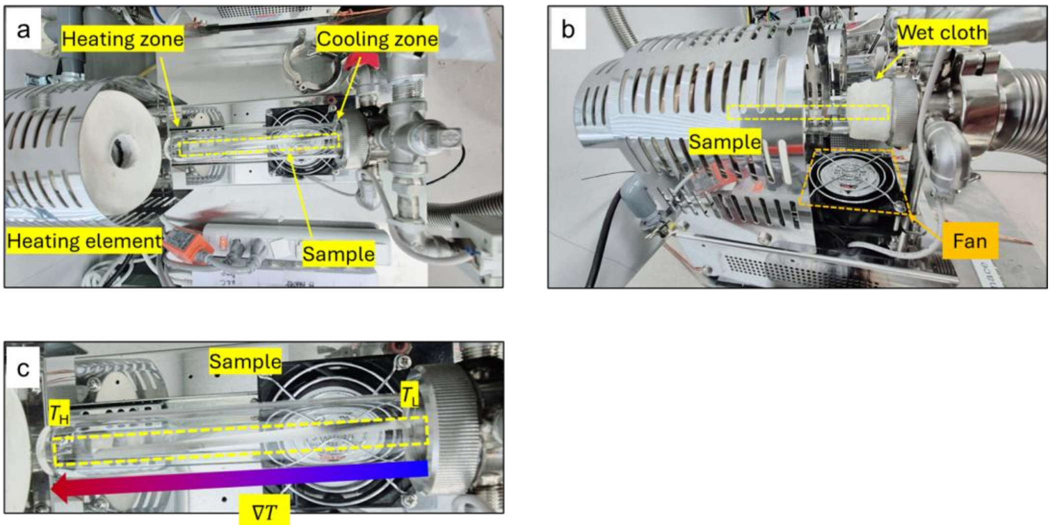

Fig. S1 | Experimental setup for fabricating functionally graded materials with controlled disorder. (a) Top view of the horizontal tube furnace. (b) Side view showing the cooling zone with a wet cloth to enhance cooling efficiency. (c) Top-view close-up of the furnace region, illustrating the natural temperature gradient imposed on the sample

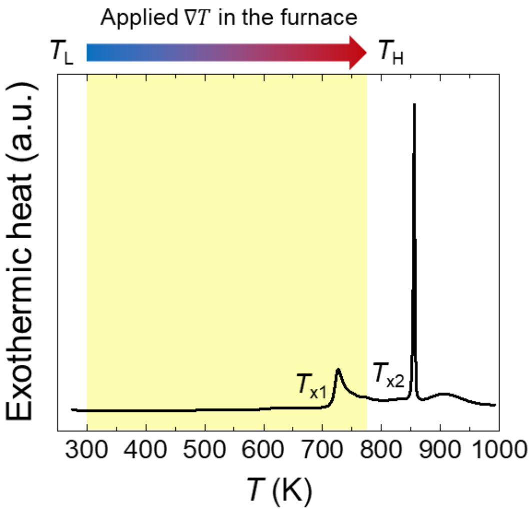

Fig. S2 | Exothermic heat measured by differential scanning calorimetry (DSC). The ramp rate was 10 K/min.

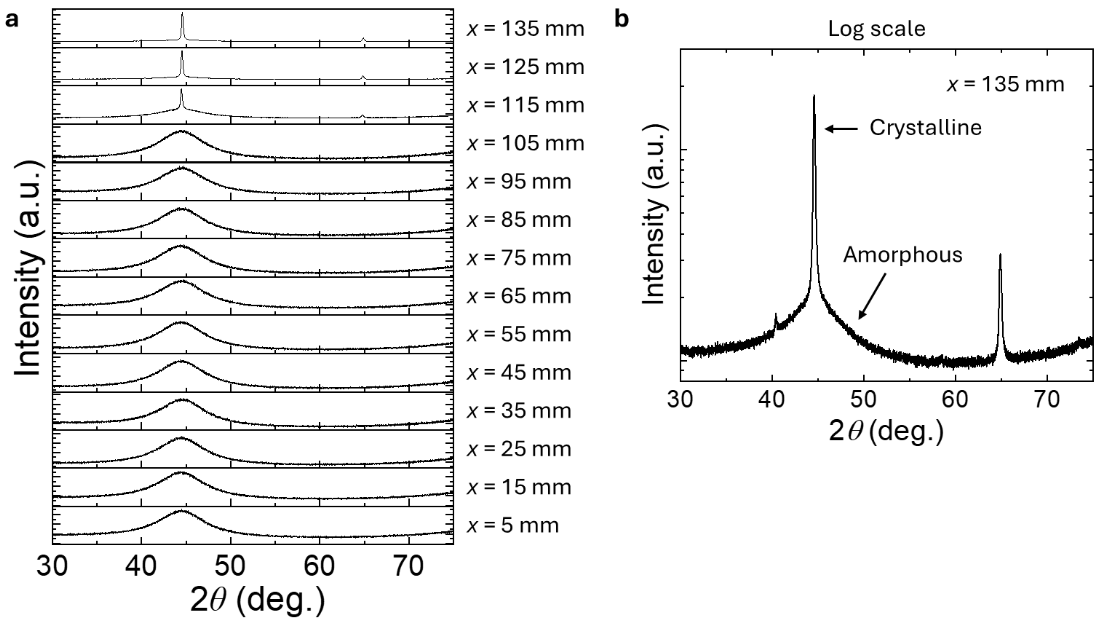

Fig. S3 | Detailed XRD data. (a) Position-dependent XRD patterns along the ribbon length (linear scale). (b) Log-scale XRD pattern at $x = 135$ mm.

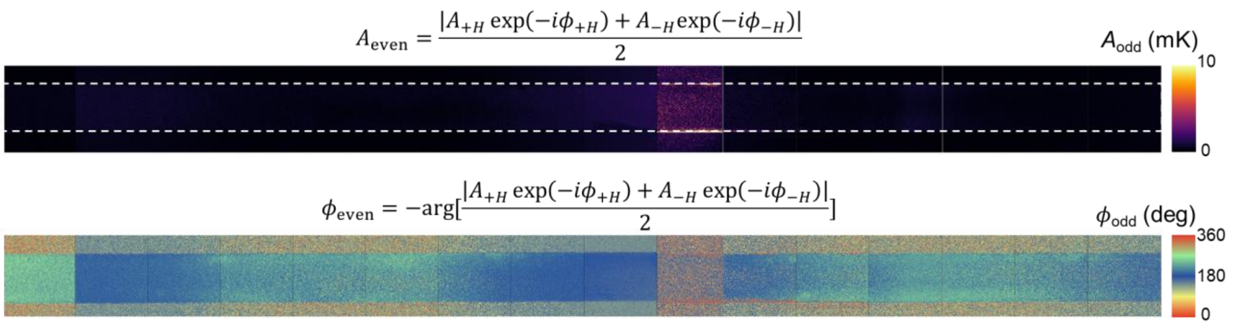

Fig. S4 | Field-even components of the lock-in thermoelectric responses. The field-even lock-in amplitude A_{even} and phase ϕ_{even} are shown. The same scales (0–10 mK for amplitude and 0–360 deg. for phase) are used for direct comparison with the field-odd component (A_{odd} and ϕ_{odd}) shown in Fig. 3. The sample boundary is indicated by white dashed lines.

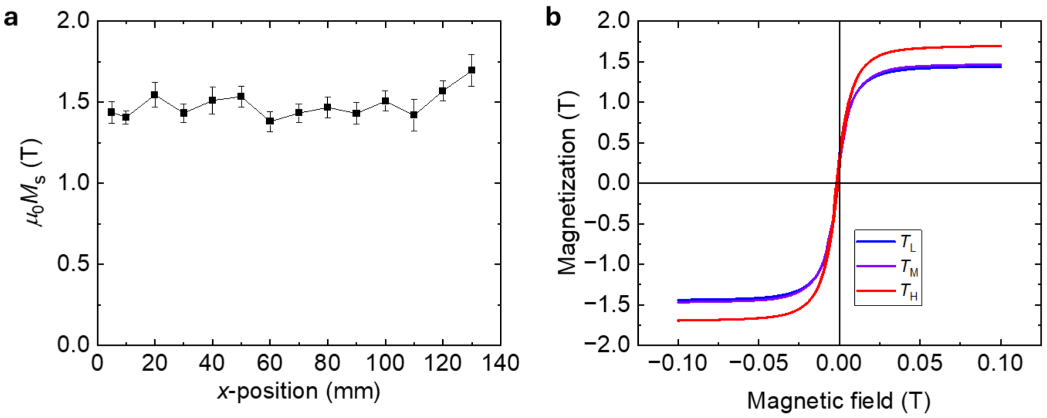

Fig. S5 | Position-dependent magnetic properties. (a) Position-dependent saturation magnetization (M_s). (b) Magnetization as a function of magnetic field for three representative positions corresponding to T_L, T_M, and T_H. All measurements were conducted at room temperature ($T = 300$ K).

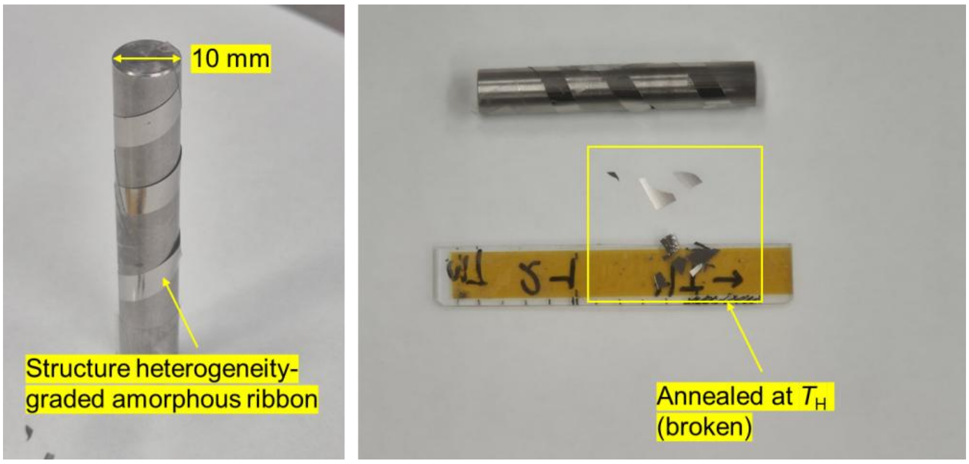

Fig. S6 | Mechanical flexibility test. The sample was attached to a curved surface with a diameter of 10 mm. The overall sample retained mechanical flexibility; however, the crystalline regions annealed on the high temperature side (T_H) broke due to brittleness during handling, specifically when detaching the samples from the double-sided tape used for electrical conductivity measurements.
